# Bioassay-Directed Isolation of Active Compounds with Antiyeast Activity from a *Cassia fistula *Seed Extract

**DOI:** 10.3390/molecules16097583

**Published:** 2011-09-05

**Authors:** Subramanion L. Jothy, Zuraini Zakaria, Yeng Chen, Yee Ling Lau, Lachimanan Yoga Latha, Lai Ngit Shin, Sreenivasan Sasidharan

**Affiliations:** 1 Biological Program, School of Distance Education, Universiti Sains Malaysia, Minden 11800, Penang, Malaysia; 2 Institute for Research in Molecular Medicine (INFORMM), Universiti Sains Malaysia, 11800, Pulau Pinang, Malaysia; Email: srisasidharan@yahoo.com (S.S.); 3 Faculty of Dentistry, University of Malaya, 50603 Kuala Lumpur, Malaysia; 4 Department of Parasitology, Faculty of Medicine, University of Malaya, 50603 Kuala Lumpur, Malaysia; Email: lauyeeling@um.edu.my (Y.L.L.)

**Keywords:** bioactive compounds, bioassay-directed isolation, natural products extraction, fractionation

## Abstract

*Background and objective*: *Cassia fistula *L belongs to the family Leguminosae, and it is one of the most popular herbal products in tropical countries. *C. fistula* seeds have been used as a herbal medicine and have pharmacological activity which includes anti-bacterial, anti-fungal, and antioxidant properties. The goal of this study was to identify compounds from *C. fistula* seeds which are responsible for anti-*Candida albicans* activity using bioassay-directed isolation. *Results*: The preliminary phytochemical screening of the plant seed revealed the presence of anthraquinones, flavonoids, saponins, tannins and terpenoids. The isolation of active compounds was carried out in four steps: multiple extractions, fractionation using column chromatography and purification using preparative thin-layer chromatography (TLC) and liquid chromatography/mass spectrometry (LC/MS). The structure of separated compounds was determined on the basis of mass spectrometry data. One compound was identified is roseanone. *Conclusions*: The MS analysis on the active fraction from seed extract of *C. fistula* confirmed the presence of roseanone with antiyeast activity.

## 1. Introduction

*Cassia fistula* L., (Leguminosae) is a semi-wild Indian Labernum ornamental tree with beautiful yellow flowers. This plant can be found in various countries in Asia, South Africa, Mexico, China, West Indies, East Africa and Brazil [1]. This plant is widely used by tribal people to treat various ailments including ringworm and other fungal skin infections [2]. *C. fistula *exhibited significant antimicrobial activity and showed properties that support its folkloric use as a broad-spectrum antimicrobial agent in the treatment of some diseases [3]. *C. fistula *plant parts are known to be an important source of secondary metabolites, most notably phenolic compounds. 

Our previous study showed that *C. fistula *exhibited good antiyeast activity against *C. albicans.* Recently, chemists worldwide have paid attention to the potential of medicinal plants as alternative sources for the isolation of novel metabolites with interesting biological and pharmaceutical properties [4,5]. The present study deals with the isolations of the active biological compound(s) from the crude methanol extract of *C. fistula*. Bioassay-directed fractionations results of crude extracts can help isolation of active compounds [6]. It provides evidence of the possible presence of several active compounds in the crude extracts [7]. In this study we used a bioautographic technique and Liquid Chromatography-Mass Spectrometry (LCMS) for the isolation and identification of antiyeast compound(s) against *C. albicans *from a crude extract of *C. fistula.*

This study is part of a larger investigation aimed at screening different plant extracts for their antiyeast activity. Furthermore, this extracts was explored for their effect on the pathogenic *C. albicans*. Within this framework, *C. fistula *seed extract was investigated in more detail. In particular, we have focused on compounds, present in methanol extracts of *C. fistula *seed, which were identified and characterized using different analytical techniques.

## 2. Results and Discussion

Isolation and identification of bioactive compounds present in a crude extract sample, has emerged as the major path of antibiotic development from natural products based antimicrobial agents besides their biological activities. Isolation and identification of bioactive compounds found in crude extracts as building blocks of new antibiotics provides unique opportunities for the development of antibiotics with new mechanisms of action and more potent drugs to treat various human ailments. The analysis of bioactive compounds in the crude extract is dramatically more complex than gene expression analysis. There is an enormous bioactive compound diversity of a plants crude extract to be covered. This presents a considerable challenge for the isolation and identification of bioactive compounds. During the last decade, LC-MS techniques were developed employing soft ionization methods like electro- spray (ESI) or photoionization (APPI) and, simultaneously, mass spectrometers have become both more sophisticated and more robust for daily use. More recently, achievements in separation sciences propose much better solutions for the separation of the complex mixtures than it was attainable before. Specifically, the advances in the preparation of monolithic columns has enabled the separation of very complex mixtures due to the high number of theoretical plates of these columns [8]. Hence in this study various methods, including LC-MS techniques, were utilized to identify the bioactive compounds with antiyeast activity from crude extract of *C. fistula *seeds.

Qualitative analysis carried out for methanolic extract of the seeds of *C. fistula* showed the presence of five major groups of phytochemical constituents and is summarized in Table 1. Phytochemical screening of the plant seed revealed the presence of anthraquinones, flavonoids, saponins, tannins and terpenoids in the *C. fistula *extract. Further purification and identification of bioactive compound were done using various chromatography and spectrometry methods as mentioned in the Experimental section.

**Table 1:** The analysis of phytochemicals in the methanol seed extract of *C. fistula.*

Test	Seed extract	Observation
Reducing Sugar	–	dark greenish
Anthraquinone	+	deep red coloration of aqueous layer
Terpenoids	+	deep red coloration
Flavonoids	+	colorless
Saponins	+	persistence of frothing
Tannins	+	dark greenish grey coloration
Alkaloids	–	dark greenish

+: Presence; –: Absence.

**Figure 1:**
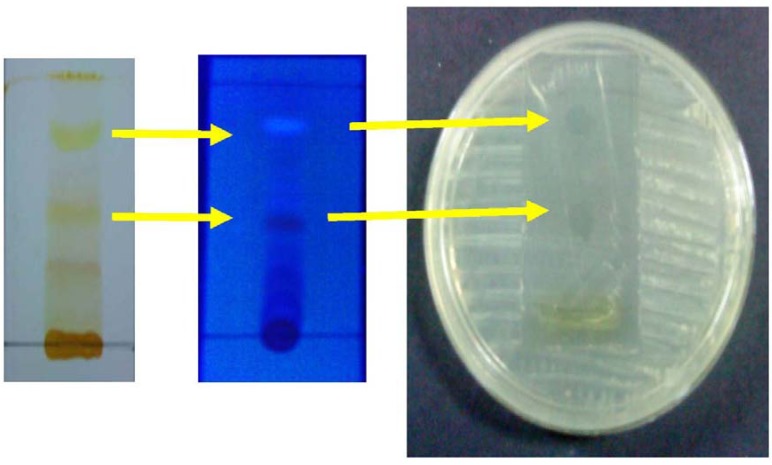
TLC bioautogram showed the band with antiyeast activity against *C. albicans *at the position of *R*_f_ 0.63.

The column chromatography was run by the above method and it yielded 16 fractions. The antiyeast activity of each fraction against *C. albicans* was measured by the disk diffusion method and the ninth fraction showed a high antiyeast activity (18 mm). Further purification was done by the preparative thin-layer chromatography, to identify the active fraction by a bioautographic technique. The thin layer chromatography eluant system (methanol:chloroform, 10:90), separated compounds over a wide range of R_f_ values. The inhibition zones produced by TLC bioautographic technique, showed the band with antiyeast activity against *C. albicans* at the position with *R*_f_ 0.63. Bioautography of TLC chromatograms worked well with *C. albicans* in this study [9]. The band, which showed antiyeast activity was also viewed under UV and showed a blue fluorescence (Figure 1).

Subsequently, the active fraction was collected by scraping off the band and then re-dissolving in methanol for the HPLC and LC-MS procedures. A number of problems arise from LC-MS protocols for bioactive profiling. Some of these problems are related to the LC separation itself, others are connected to the MS detectors used. In general, mass spectrometers are able to detect a large number of the compounds simultaneously, even if these are co-eluting. Selectivity is gained by identification and quantification of the compound’s specific molecular masses and fragmentation pathways. However, mass spectrometers can only detect ions, with the result, that any quantification demands that there is perfect ionization of all compounds throughout the chromatogram. Unfortunately, this is not always the case. It is well known among mass spectrometrists, that one compound can hamper the ionization efficacy of another, even if these are chemically related and co-elute at the same time. Therefore, ultimate separation is needed to ensure robust quantification, especially for very complex mixtures consisting of hundreds of components to be analysed and quantified [10]. However the active fraction isolated from crude extract of *C. fistula *seeds extract consisted of less than 10 compounds providing considerably less challenge for the isolation and identification of bioactive compounds in this study. 

**Figure 2:**
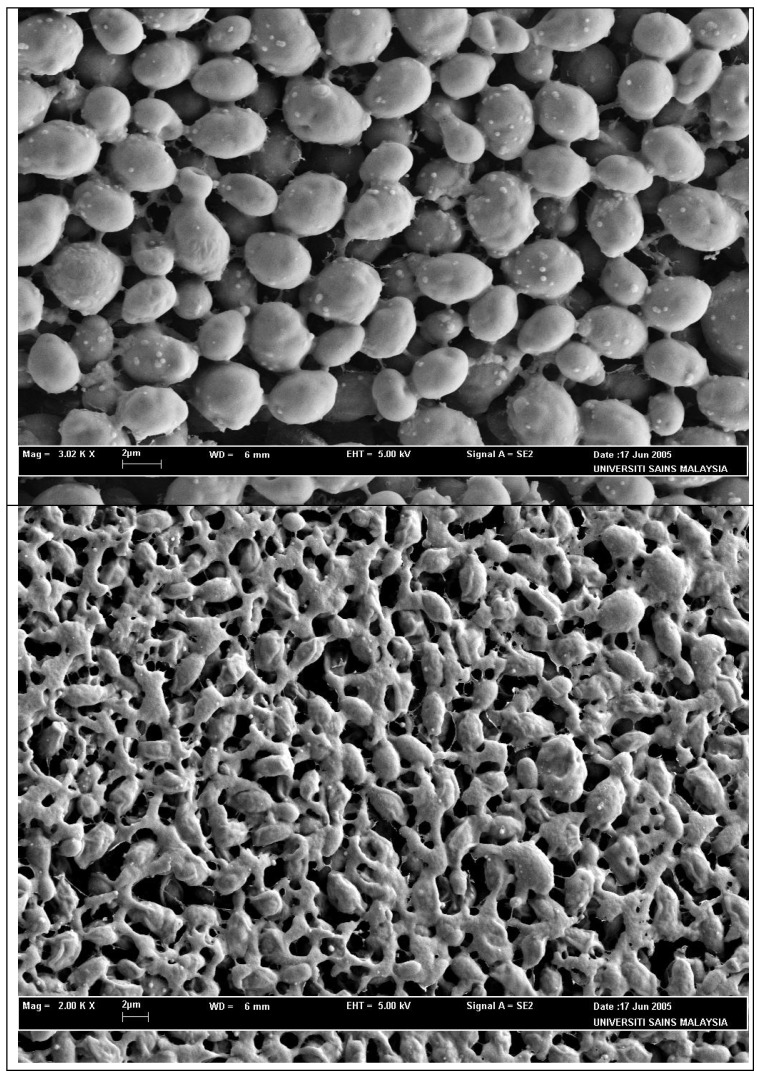
Scanning electron microscope photomicrograph of the untreated (**a**) and extract-treated (**b**) cells of *Candida albicans*.

The active fraction, therefore, holds the most promise for further work, as far as the antimicrobial components are concerned. The active fraction, showed a dose-dependent antimicrobial activity against the yeast tested with zones of inhibition that ranged from 12 to 20 mm in size. The clearance zone produced by the commercial antibiotic (miconazole nitrate) disk was larger than those produced by the active fraction disk (Table 2). The solvent-only negative control disk produced no clearance zone (Table 2). Figure 2 shows the SEM photomicrographs of the untreated and active fraction-treated cells of *C. albicans*, at 36 hours of exposure to the active fraction of *C. fistula *seed. Untreated cells (Figure 2a) showed many oval and smooth cells in appearance with some at a budding stage. The cells treated for 36 hours (Figure 2b) showed severe alterations and completely collapsed and lysed yeast cells. It was assumed that at this phase, the cells had lost their metabolic functions totally. The wide strong anticandidal activity of active fraction may provide clinically useful leads.

**Table 2:** Zone of inhibition of *Cassia fistula* seeds extract, active fraction and commercial antibiotics against *Candida albicans.*

Microorganism	Zone of inhibition (mm)		
	Crude extract	Active fractions	Miconazole nitrate
***Candida albicans***	21	250 µg/mL–18	22
		500 µg/mL–20	
		1000 µg/mL–22	

**Figure 3:**
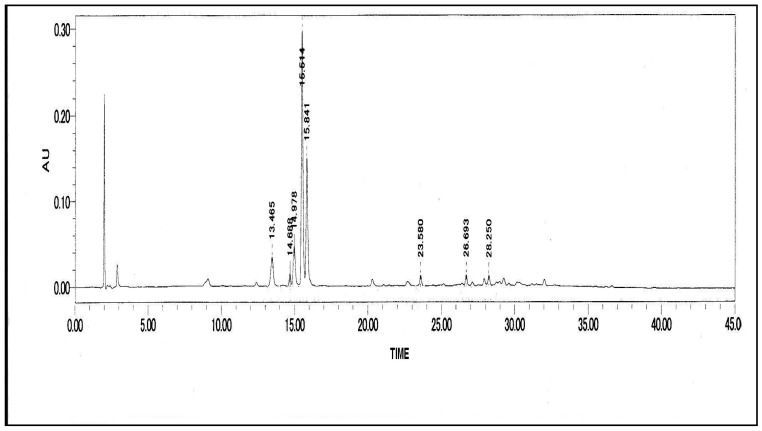
HPLC chromatogram of active fraction isolated from seeds extract of *Cassia fistula.*

**Figure 4:**
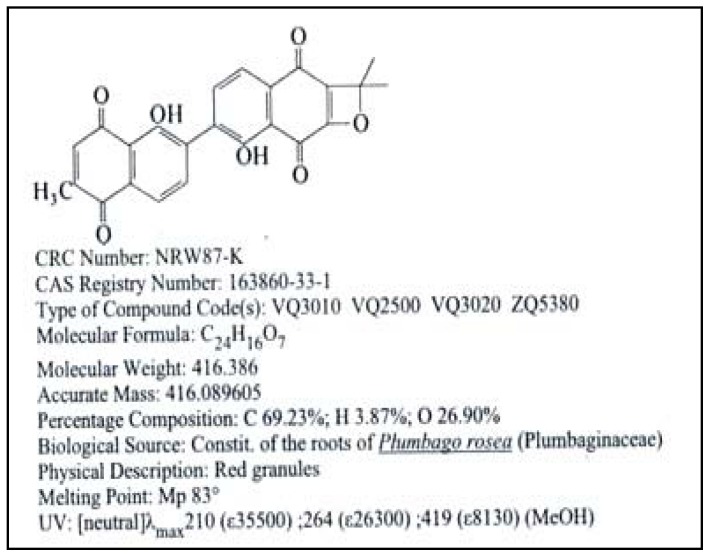
Molecular structure of Roseanone (C_24_H_16_O_7_), mol wt = 416.386 [11].

HPLC analyses of the active fraction showed the presence of eight peaks with one distinct peak, making identification of compounds very difficult (Figure 3). Each of the peaks may represent one or more compounds present in the active fraction. Typically chromatographically separated peaks would contain a single major component. Initial HPLC and LC/MS tests on this distinct peak indicated the presence major bioactive compound with antiyeast activity in the active fraction isolated from crude methanolic extract of *C. fistula *seeds. The MS analysis on this component was subsequently confirmed it as roseanone (Figure 4). Roseanone is a class of natural phenols based on the C6-C4 skeleton. 

## 3. Experimental

### 3.1. Plant Collection

The pods of *C. fistula *were collected from various areas around Universiti Sains Malaysia, Penang in November 2009 and authenticated by the botanist of the School of Biological Sciences at Universiti Sains Malaysia where the herbarium sample was deposited. The sun-dried pods were cut open and the seeds removed from the pods. The seeds were then washed thoroughly and rinsed with tap water and dried in oven at 60 °C for three to four days. Then the dried seeds were homogenized to a fine powder and stored in airtight bottles. 

### 3.2. Solvent Extraction

One hundred fifty grams (150 g) of dried powder was extracted with 80% methanol (v/v, 400 mL) for one week. Then it was filtrated through filter paper and the entire extract of *C. fistula *seeds was evaporated under reduced pressure using a rotary evaporator. The extract then evaporated at 50 °C in oven to give a paste form. Then it was sealed in Petri dishes and stored at room temperature for further studies. 

### 3.3. Preliminary Phytochemicals Screening

Pytochemical tests were studied or analysed on the methanolic extract of the powdered form of the seed sample using standard qualitative methods as described by Edeoga *et al*. [12] and Harborne [13].

### 3.4. Microorganism

*Candida albicans *(local isolate) was used as the test organism and was obtained from the laboratory stock culture. The yeast was cultured in Sabouraud Dextrose agar at 30 °C for 24 h. Stock cultures were maintained at 4 °C on the slopes of tryptic soy broth (BBL, Cockeysville, MD, USA) amended with 5 g/L yeast extract (Oxoid, Nepean, ON, Canada) and 15 g/L agar (BDH, Toronto, ON, Canada).

### 3.5. Column Chromatography

The *Cassia fistula* seeds extract were further subjected to column chromatography. An open glass column (150 by 200 mm) was packed with silica gel (Merck, Darmstadt, Germany, 0.063 to 0.200 mm). The column was eluted with 150 mL methanol-chloroform (10:90, v/v). The fractions were collected in 5 to 10 mL portions depending on the visible changes in the colorful bands running out of the column. Based on TLC profile of fraction the second column chromatography was performed to further dissociate a few fractions. The eluted fractions (5 mL) were vaporized in the oven at 60 °C. Then each fraction again re-dissolved in the methanol and test for anticandidal activity against *Candida albicans* using the disc diffusion method [14]. 

### 3.6. Antimicrobial Activity

The antimicrobial activity of the extract of the active fraction was determined following the method described by Miles and Amyes [14], with slight modifications.

#### 3.6.1. Disk Diffusion Technique

The test microbes were removed aseptically with an inoculating loop and transferred to a test tube containing 5.0 mL of sterile distilled water. Sufficient inoculum was added, until the turbidity equaled to 0.5 McFarland standards (BioMerieux, Marcy Petoile, France). One mL of the test tube suspension was added to 15–20 mL of nutrient agar, before setting aside the seeded agar plate (9 cm in diameter) to solidify for 15 minutes. Three Whatman's filter paper No. 1 disks of 6 mm diameter were used to screen the antimicrobial activity. Each sterile disk was impregnated with 20 µL of the fraction (corresponding to 1.00, 0.50, and 0.25 mg/mL active fraction); Miconazole nitrate, a commercial antibiotic (30 µg/mL, as positive control), and methanol the solvent used for extraction (v/v) (20 µL, as negative control) was placed on the surface of the seeded plates [15]. The negative and positive controls were used in this study to carefully verify that the supposed effects of the extract are produced only by the extract itself. The plates were placed at 4 °C for two hours, followed by incubation at 37 °C, for 24 h and examined for zones of growth inhibition, which were expressed in millimeters (mm). Each test was performed in triplicate and repeated twice.

### 3.7. Isolation of Antiyeast Substances from Crude Extract of C. fistula

The fraction which showed a high antiyeast activity was purified by preparative thin-layer chromatography (TLC) (silica gel 60F_254_ [0.2 mm thick]; Merck). Thin-layer chromatography plates, loaded with crude extracts, were developed with a chloroform-methanol (90: 10 [v/v]) solventsystem. After the plate was air dried, a silica gel band which showed antiyeast activity against *C. albicans* atthe position of *R_f_* 0.61 was collected by scraping off the band, and eluted with methanol. The inhibition zones produced on the TLC plates were visualized by the bioautographic technique [9].

#### 3.7.1. High-Performance Liquid Chromatography (HPLC) Separation

An amount (0.3 mg) of the fraction which showed a high antiyeast activity was repeatedly run on an analytical HPLC column using a Constametric III pump (LDC/Milton Roy) and a spectromonitor D UV detector. The solvent was MeOH-water (85:15), the flow rate 1 mL/min, UV 220 nm, which gave eight fractions with a prominent peak at 15.514.

#### 3.7.2. Identification of Compound

The prominent peak compound with 15.514 retention time was analyzed using liquid chromatography/mass spectrometry (LC/MS) with a quadrupole ion trap MS (Bruker Esquire LC/MS, Billerica, MA, USA). The column used was a Symmetry (Waters) C_18_ column (250 × 4.6 mm). A 25-mL sample volume was injected using the system’s autosampler. Solvent A contained 5% formic acid in water, and solvent B consisted of HPLC-grade methanol. The UV response during LC/MS was monitored at 360 nm, the highest absorbance wavelength for each set of components as determined from prior HPLC studies. The LC/MS was operated in the positive-ion mode using the electrospray ionization (ESI) source and the manufacturer’s recommended operating conditions. 

#### 3.7.3. Effect of the Active Fraction on *C. albicans * Cells by Scanning Electron Microscopy (SEM) Study

One mL of the *C. albicans *cells suspension of the concentration of 1 × 10^6^ cells per mL was inoculated on a Sabouraud dextrose agar plate and then incubated at 30 °C for six hours. Ten μL of the active fraction, at a concentration of 1.00 mg per mL, was dropped onto the inoculated agar and further incubated for another 36 hours, at the same incubation temperature. A methanol treated culture was used as a control. A small block of yeast containing agar was withdrawn from the inoculated plate, at 36 hours and fixed for SEM [16].

## 4. Conclusion

In conclusion, a combination of mass spectrometry, chromatography, and biological testing was particularly useful for the rapid characterization of unknown active compounds of an extract. It is a great help in isolation and structure determination. The MS analysis on the active fraction confirmed the present of roseanone with antiyeast activity.
